# Cervical, Mediastinal, and Pericardial Disseminated Coccidioidomycosis: The Importance of Source Control

**DOI:** 10.1155/crdi/3448219

**Published:** 2026-04-05

**Authors:** Leah Grant, Seth Assar, Farhan Taghizadeh, Joann Hutto, Neil M. Ampel

**Affiliations:** ^1^ Department of Infectious Diseases, HonorHealth, Scottsdale, Arizona, USA; ^2^ Department of Pulmonology and Critical Care Medicine, HonorHealth, Scottsdale, Arizona, USA; ^3^ Department of Otolaryngology, HonorHealth, Scottsdale, Arizona, USA; ^4^ Department of Pathology, HonorHealth, Scottsdale, Arizona, USA; ^5^ College of Medicine, University of Arizona, Tucson, Arizona, USA, arizona.edu

**Keywords:** *Coccidioides*, coccidioidomycosis, invasive fungal infection, mediastinal infection, neck abscess, pericarditis

## Abstract

A 47‐year‐old healthy man developed disseminated coccidioidomycosis involving his mediastinal and cervical lymph nodes that was complicated by pericardial involvement despite continued fluconazole for primary pulmonary infection. Although he subsequently received an aggressive antifungal regimen with voriconazole and liposomal amphotericin B, he did not clinically improve until he underwent major debridement and drainage, highlighting the need for source‐control procedures in some cases of severe disseminated coccidioidomycosis.

## 1. Introduction

Coccidioidomycosis is caused by the dimorphic fungi *Coccidioides* spp., which are endemic to the western United States as well as Mexico and parts of Central and South America. Infection most commonly occurs through inhalation of airborne arthroconidia, which results in symptomatic pulmonary infection in 40% of patients ([1]). Of those who become infected with *Coccidioides*, approximately 1% of patients develop extrathoracic disseminated infection ([2]). The most common sites of dissemination include bones, skin, lymph nodes, and the central nervous system. Risk factors for disseminated infection include pregnancy, suppression of the cellular immune system, and certain genetic backgrounds, including African and Filipino ancestry ([3]). Once dissemination occurs, gaining control of the widespread fungal infection can be challenging.

## 2. Case Presentation

A 47‐year‐old man of African ancestry presented to the emergency department (ED) with fever, cough, night sweats, and chest pain associated with patchy left perihilar hazy opacities on a plain chest radiograph. He was initially prescribed cephalexin and azithromycin and, when coccidioidal serologies returned positive for IgM and IgG by EIA, the patient was started on fluconazole 400 mg daily.

Four months later, the patient developed back pain and swelling of the left supraclavicular area while still taking fluconazole. A computed tomography (CT) scan of the chest revealed a left apical pulmonary nodule with thoracic lymphadenopathy as well as lytic lesions of the thoracic spine. An ultrasound‐guided aspirate of the left supraclavicular neck mass obtained 15 mL of purulent fluid that grew *Coccidioides*. Serum coccidioidal complement fixation titer was ≥ 1:256. The patient was subsequently admitted to the hospital, fluconazole was discontinued, and voriconazole and liposomal amphotericin B were initiated. Further imaging revealed osseous lesions throughout the spine and pelvis, discitis and osteomyelitis of the seventh and eight thoracic vertebrae, and cervical and mediastinal lymphadenopathy. A CT scan of the neck demonstrated a 6.5‐cm mass at the left cervical base that extended into the superior mediastinum. The patient underwent two additional percutaneous drainage procedures of the left neck abscess, and he was discharged on oral voriconazole 4 mg/kg twice daily and liposomal amphotericin B 3 mg/kg three times each week. Given the finding of disseminated coccidioidomycosis, the patient was evaluated for immunocompromising conditions including HIV infection and diabetes, and tested negative.

**FIGURE 1:**
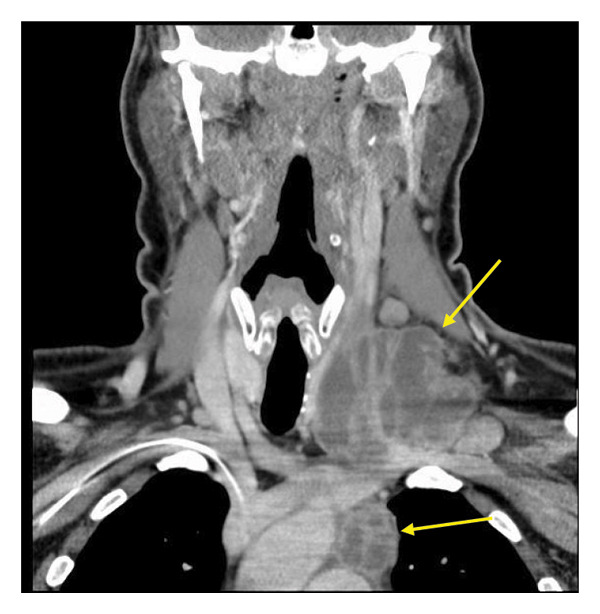
Coronal CT image of the large left‐sided neck abscess extending into the superior mediastinum.

**FIGURE 2:**
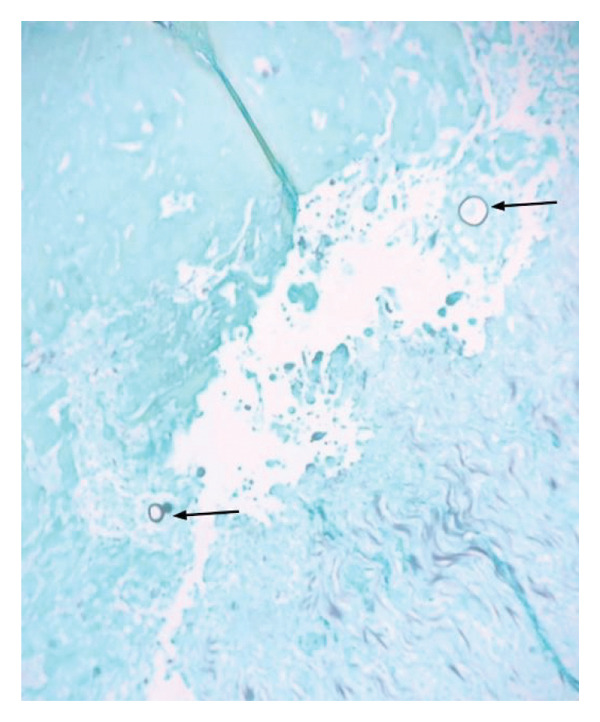
Pericardial tissue with likely coccidioidal spherules (arrows), GMS stain (400x).

Over the next 2 months, the patient worsened and was readmitted because of chest pain and dyspnea. He continued to receive liposomal amphotericin infusions as well as voriconazole and demonstrated therapeutic voriconazole drug levels on multiple occasions. He was found to have progressive enlargement of the left neck abscess with mass effect on the internal jugular vein (Figure 1) and a new large pericardial effusion with features of cardiac tamponade. The patient underwent an emergent pericardiocentesis and pericardial window procedure with 900 mL of pericardial fluid drained. Pericardial tissue demonstrated coccidioidal spherules (Figure 2). Due to progressive enlargement of the left neck abscess with impending encroachment upon the aortic arch, the patient underwent an incision and debridement of the large deep neck space abscess with placement of a drain. Operative cultures grew *Coccidioides*.

**FIGURE 3:**
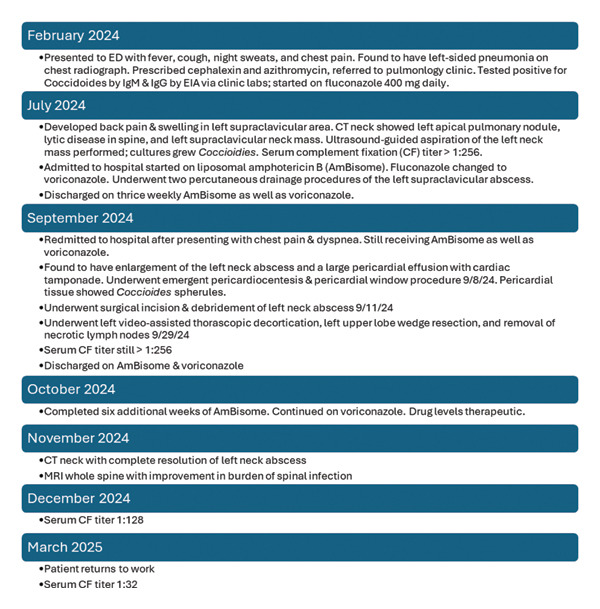
Patient clinical timeline.

The postoperative course was complicated by ongoing dyspnea and the development of a left‐sided pleural effusion. The patient ultimately underwent a left‐sided video‐assisted thoracoscopic decortication with removal of a necrotic lymph node and a necrotic portion of the left upper lung as well as lysis of adhesions and pleural lavage. An additional incision and debridement procedure of the left neck abscess was performed as well. After these procedures, voriconazole and liposomal amphotericin B were continued, and the patient subsequently clinically improved. After 3 months, the left neck abscess had resolved, and his drain was removed. After 5 months, the patient was able to return to work. His coccidioidal complement fixation titer had decreased to 1:32 by that time (Figure 3). He continues to receive antifungal therapy indefinitely.

## 3. Discussion

This case is unusual because of the development of extrathoracic disease while on fluconazole therapy, pericardial involvement as a consequence of cervical and mediastinal abscesses, and the need for extensive surgical debridement in addition to antifungal therapy to obtain control of the infection. There are several possibilities for the failure of antifungal therapy in this case. The patient’s left‐sided coccidioidal neck abscess was initially managed with percutaneous drainage, and it was not until 2 months after the discovery of the neck abscess that the patient underwent surgical debridement. This lack of an initial definitive source‐control procedure for the abscess likely contributed to further mediastinal spread of infection as well as failure of antifungal therapy.

Despite the escalation of antifungal therapy to voriconazole and liposomal amphotericin B, the patient’s mediastinal infection continued to progress, and he ultimately required a thoracic decortication procedure as well as partial lung resection and removal of necrotic lymph nodes. He required an additional surgical debridement of the left neck abscess as well. Following the aforementioned procedures, he began to improve clinically. Our case highlights the critical importance of source control in this case of disseminated coccidioidomycosis and that the escalation of antifungal therapy is unlikely to succeed in the presence of undrained foci of infection.

Another possible reason for antifungal failure is that systemic levels of the drug were too low or that the *Coccidioides* was resistant. No specific data for this patient are available for either case, but there has been concern that *Coccidioides* frequently manifests relatively high minimum inhibitory concentrations to fluconazole, suggestive of decreased susceptibility compared to other triazole antifungals and amphotericin B ([4]). In addition, in the only comparative antifungal trial, itraconazole was associated with an improved outcome in skeletal coccidioidomycosis ([5]). Based on these observations, a change should be considered in patients failing to respond to initial treatment with fluconazole to a mold‐active triazole, and in severe cases, by the addition of amphotericin B to triazole therapy, as was done in this case. An additional contributor to failure of antifungal therapy may have been the lack of penetration of the drug(s) into necrotic lymph nodes and necrotic tissue.

Pericardial involvement with *Coccidioides* is a rare but well‐described phenomenon ([6–9]). In this instance, it appeared to be due to direct extension from the cervical abscess with proximate exposure to infected mediastinal and hilar lymph nodes. In a review of 24 cases of pericardial involvement ([10]), most patients recovered with pericardiectomy or pericardial drainage combined with antifungal therapy, as occurred here.

## 4. Conclusion

This case emphasizes the need for source control in some instances of extrathoracic coccidioidomycosis. In this case, reducing the burden of coccidioidal infection appeared to allow the patient to control the infection with the assistance of appropriate antifungal therapy. There are other similar reports ([11–13]), and such an approach should be considered in any patient with extrathoracic coccidioidomycosis failing appropriate antifungal therapy who has lesions amenable to surgical debridement.

## Author Contributions

Leah Grant and Neil M. Ampel wrote and edited the manuscript. Farhan Taghizadeh, Joann Hutto, and Seth Assar participated in the patient case and reviewed the manuscript. The corresponding author is Leah Grant.

## Funding

The authors of this publication did not receive specific funding, and the work was completed as part of employment under HonorHealth.

## Conflicts of Interest

The authors declare no conflicts of interest.

## Data Availability

Data sharing is not applicable to this article as no datasets were generated or analyzed during the current study.
